# Parliament and the Professions

**Published:** 1919-04-19

**Authors:** 


### PARLIAMENT AND THE PROFESSIONS.

### Recognition of Doctors' War Service.

Mr. Churchill stated that the valuable services of physicians and surgeons who have given voluntary service in war hospitals controlled by the War Office have not been overlooked. It is not possible to prepare a list of all these gentlemen, but the War Office have a comprehensive list of those who have been recommended for recognition in connection with the "Honours Gazette" of June 3, 1918.

### Military Hospitals.

Sir J. Hope asked the Secretary for War whether the New Zealand Force have offered to hand over to the War Office three hospitals, each having 2,000 beds, and whether he would consider the closing down of small military hospitals, both in the interests of economy and in order that better effect may be given to the scheme of vocational training for civil life among walking patients in hospitals by their concentration in large hospitals. Mr. Churchill said no such offer had been received. Inquiry had been made as to when the hospitals would be vacated in order that the War Office may have an opportunity of stating whether it is desired to take them over or not. The policy is to close down the smaller hospitals and concentrate in the larger hospitals. Mr. Churchill stated that the 1st Northern General Hospital, Newcastle-upon-Tyne, is to be closed as soon as the Royal Victoria Infirmary can take the patients. The Northumberland War Hospital is to be closed by the beginning of May. The evacuation of beds occupied by military patients in civil hospitals is progressing as speedily as circumstances permit.

### V.A.D. Hospitals.

Captain Guest informed Sir Samuel Scott that, including those provided in hospitals by the Red Cross and those provided by private individuals independently, the total number of beds during the war was well over 80,000. He had no means of estimating what additional cost, beyond the grants made from Army funds to these hospitals, would have fallen upon the public if these beds had not been so provided, but the amount would have been large. The assistance rendered is very fully appreciated.

### War Gratuity for V.A.D. Nurses.

Mr. Forster stated that a war gratuity will be given to Voluntary Aid Detachment nurses in Army employment. It is hoped to make an announcement on the subject shortly.

